# On the Pathology of Fever

**Published:** 1826-06

**Authors:** Wm. Stoker

**Affiliations:** senior Physician to the Fever Hospital and House of Recovery, Cork-street, Dublin.


THE LONDON
### Medical and Physical Journal.

No. 6 of vol. lv.] JUNE, 1826. [No. 328.

For many fortunate discoveries in medicine, and for the detection of numerous errors, the world is indebted to the rapid circulation of Monthly Journals; and there never existed any work, to which the Faculty, in Europe and America, were under deeper obligations, than to the Medical and Physical Journal of London, now forming a long, but an invaluable, series.—RUSH.

### ORIGINAL COMMUNICATIONS,
### SELECT OBSERVATIONS, &c.

Art. I.—*On the Pathology of Fever.* By Wm. Stoker, m.d. senior Physician to the Fever Hospital and House of Recovery, Cork-street, Dublin.

The new doctrines of fever, which I adverted to as a branch of solidism, at the conclusion of my last letter in this Journal,* in as much as they attributed fevers generally to inflammation, or altered structure of some vital part, and suggested blood-letting as their common remedy, were directly opposed in principle to that theoretic division of fevers into idiopathic and symptomatic, which I have ever deemed natural and useful in practice. It therefore became my duty, as physician to the Fever Hospital, to examine the arguments closely on which these doctrines† were promulgated and maintained; more especially as they

* See Number for March 1826, page 183.

† These doctrines, as successively adopted by Drs. Clutterbuck, Armstrong, Mills, and M. Broussais, may be found detailed in the works of each of those authors; and the favourable reception which they generally met with, may be judged of from the Reviews of these works, as they appeared soon after their publication.

That a strong counter current in public opinion has lately commenced, may, I think, be inferred from the tenor of the articles, of late, in many of those Journals where these works were once noticed with approbation. In an able Essay by Dr. Gerson, in the Magazine of Foreign Literature, &c. &c. at Hamburgh, for July and August, 1824,[a] it appears that this counter current had commenced earlier, and more remarkably, in Germany than in Great Britain; but, in the first article of the first Number of the new Journal of Medicine, published at Edinburgh in the beginning of the present year, the return to more correct pathological views than have been taken for some time in this country, may be distinctly perceived. On a future occasion, I hope to avail myself of the great increase of

[a] "Magazin der Ausländischen Literatür der gesammten Heilkunde, &c. von Dr. G. H. Gerson, und Dr. Nichol, Henri Julius. Juli, August, 1824.—Art. 2. *Pathological Observations. Part I. on Dropsy, Purpura, and the Influenza of* 1822 *and* 1823, &c. &c. By William Stoker, Dublin, 1824.

were said to have been deduced from actual observation made in the course of diseases, or on the appearances of parts discovered after death.

The process of the examination which I accordingly made, through the opportunities offered to me in the largest and most fully occupied Fever Institution yet established, may be seen detailed in the Reports I published from that Institution, and in a separate Essay on Fever, printed at London in 1814.

In the three earliest of these Reports, evidence was adduced in favour of the division in question, showing, from several hundred cases taken without selection, that the same tendency still continued, in idiopathic and typhoid fevers, to terminate favourably or fatally on certain critical days, as had been observed to characterise them by Hippocrates.* And, in my Report for the year 1811, I added facts to prove that fevers, subject to such periodical revolutions, were frequently the consequence of exposure to contagion or infection.

The Appendix† to my Essay on Fever, however, contains the result of a trial of the merits of these doctrines, which promised to contribute more to a satisfactory decision of the question than any of the previous investigations, both on account of the extent of the comparison instituted between the opposed modes of treatment, and the faithfulness of the records from which it was deduced. But, previously to the brief extracts from this document, showing the result which I intend to add, I should observe, that, after much reflection on the subject, I feel convinced that if, in making this comparison, all those mixed cases of typhous and inflammatory fevers, in which blood-letting may have been too cautiously withheld on the one hand, and, on the other, all of the same kind, or those of actual inflammation, in which that remedy must have been beneficially employed, could be separated, the results of the numerical calculations would have been much more decisively against the

---

information which I have derived from Dr. Gerson's Essay; and must regret that, for the present, I must confine myself to the expression of my satisfaction that my work on Dropsy has been deemed worthy of so much of his attention. But, however I may wish to conciliate further his favourable opinion, I beg to explain that my views in that work were intended not by any means to overturn the pathology deduced from altered structure, but to relate facts which might suggest certain additions from the too generally exploded humoral pathology, that would render the systems now in vogue more perfect. And, though I hail the proof given in the new Journal of Medicine and Surgery of Edinburgh, by Dr. Brown, of the return of public opinion to sounder pathology, yet I cannot perceive with satisfaction that my humble, but constant, efforts in support of these tenets which he now upholds, have been unnoticed by him.

* See Collections, &c. &c. on the Effects of Sol-Lunar Influence in Fevers, by Francis Balfour, M.D. &c. &c.—London, 1816. *Preface*, page 8, 9, 10.

† See Extract of Letter from the Physicians of the Fever Hospital, &c. Cork-street, to the Managing Committee.

practice in purely typhoid fevers, than they now appear. For Dr. Mills, then one of my colleagues, who, though he had previously much experience in the treatment of fever, adopted Dr. Clutterbuck's views, with certain modifications, and practical blood-letting, in a very large proportion of cases,—at times nearly with all his patients, in the Cork-street Hospital. But, by the other physicians, that remedy was rarely employed, and the estimate of the value of repeated blood-letting in fever indiscriminately, compared with other modes of practice, (modified as just now suggested,*) still further confirmed my previous opinions. I feel it due, however, to Dr. Mills to add, that, previous to his publication on the subject, I would not have ventured on general blood-letting in mixed cases of inflammatory and typhoid fevers, to the extent which I have employed it since, (sometimes with advantage,) for the relief of coexisting or supervening symptoms of inflammation. It appears, however, from the document just referred to as "the final result, that, among the patients treated by blood-letting, the proportion of deaths to recoveries was as one to $11\frac{49}{55}$; and among those treated according to the more ordinary methods, as one to $12\frac{101}{110}$; a proportion differing from the former in no small degree, and justifying the conclusion that the treatment of fever by small and repeated general detractions of blood, is either of little efficacy or injurious."

Dr. Mills's patients have remained in the hospital, on an average, $16\frac{3}{5}$ days; the patients of the other physicians, $17\frac{2}{3}$ days; a difference very slight, and, though apparently in favour of blood-letting, yet most probably arising, not from this practice, but from Dr. Mills having under his care a much larger proportion of male patients than the other physicians; "and these patients, from obvious reasons, are less disposed than the females to remain unnecessarily in the hospital after their recovery."†

The following appears to me to be a very natural division of continued fevers, being simple and applicable to practical purposes:—1st, Idiopathic fevers, comprehending those of spontaneous and contagious origin; 2d, inflammatory fevers; and, lastly, those in which two kinds coexist or mingle together; a division, though sanctioned by the first nosological authorities, by no means unobjectionable, but adopted here for the reasons already assigned, being a more comprehensible part of the labyrinth in which a medical observer is involved by the theories of every age, to whom we may generally apply what Hippocrates

---

* See Dr. Brown's Essay on the Controlling Power of Remedies in Fever.—Edinburgh, 1815.

† See Treatise on Fever, published at London by me in the year 1815; page 193 and 4.

said of the lectures of his day, "Unusquisque suæ orationi testimonia, et conjecturas addit,—vincit hic, modo ille, modo iste, cui potissimum lingua volubilis ad populum contigerit." From such perplexity the medical prescriber can only pass securely by constant attention to the phenomena of nature."

The cases admitted into this hospital belong, in general, to either of the first or third parts of this division; their relative proportions varying much at different seasons of the year, and in different epidemics. Cases belonging to the second part also occasionally get admission, (though not strictly those for whom the institution was intended;) it being sometimes advisable to remove persons labouring under inflammatory, and even other diseases, from situations where they are exposed to all the circumstances favourable to the generation of fever and of contagion.

The characteristics of the first class of fevers, very generally, are sudden prostration of strength; failure of mental and bodily power; suspension or disorder of the secretions; irregular vascular and pulmonary action, producing both inequality in the distribution of blood and of temperature in different parts of the system, and an uniform tendency to perform a certain succession of salutary or morbid changes, in definite periods. The distinguishing features and local origin of the second class, or inflammatory fevers, are well marked and generally known; but, when their symptoms mingle, in epidemics, with those of typhoid or idiopathic fevers, the importance is commensurate with the difficulty of ascertaining their relative degrees, so as safely to decide on the means to be employed.

The opinions on which this division rests, that typhus and synochus are not essentially inflammatory, but that, in their simple forms, they are diseases of debility through their whole course; and that the excitement, so observable in their early stages, is constitutional reaction, accord with my experience.

Morbid anatomy, therefore, does not appear to me to warrant the conclusions which those who hold the opinion of typhous fever being an essentially inflammatory disease, have deduced from it. I have in some instances observed the same partial turgescence of vessels which they report, and likewise signs of inflammatory action in various parts of the bodies of those who died of fever: the former, however, I believe, is by no means a mark of previous inflammation; and I could generally trace the commencement of inflammatory action, which produced the latter appearance, to local disease, which preceded or supervened on fever, sometimes at late stages; but in several cases, where I had witnessed the highest degree of febrile excitement before death, no such signs of turgescence, or of inflammation, were observable on dissection.

In support of the foregoing statements, I was happy to be able to add, in the same Report, the highly respectable testimony which Mr. Kirby's answers to my queries on the subject enabled me to bring forward. Besides the unquestionable qualifications of so accomplished an anatomist, and his numerous opportunities for examining the bodies brought to his theatre for dissection, his opinion, as expressed in the following extract from his letter, had the peculiar advantage of being deduced from observations that were not influenced by preconceived theory, or by cases selected for particular views.

"In the midst," he says, "of all the discussions relative to topical congestion, it was impossible, however, not to remark the singular want of accordance between the prevalent opinions and local appearances. The brain, so constantly supposed to be the seat of inflammation, rarely exhibited the characters indicative of such a state. In some instances, this organ was much paler than usual; in a very few, amongst a great number of dissections, was there any evidence of sanguineous or serous effusion." And, after recounting his observations on all the other vital organs, he thus concludes:—"In short, in a great majority of cases, so little did any particular organ seem to suffer, that I have wondered what could have been the cause of death."

With the foregoing statement, the answers of Dr. Macartney, anatomical professor of Trinity College, Dublin, to queries of Dr. Barker on the same subject, are coincident, and appear to me further to corroborate the opinions that typhous and inflammatory fevers are distinct. I may, therefore, be allowed to add the following extract from Dr. Barker's Report for the years 1817 and 18, as published in the second volume of Transactions of the Irish College of Physicians, page 574.

"He (Dr. Macartney) informs me, that, 'having reviewed his notes on the anatomical examination of persons who have died of typhous fever, he can state, as the result of his experience, that morbid appearances in typhous fever are not those of common visceral inflammation;' and, having stated more fully the actual observations on which this opinion was founded, he concludes thus:—

"'Two facts deserve to be recollected:—1st. That the duration of general fever and visceral inflammation are not the same; 2d. That internal inflammations are very common in hot-blooded animals, but idiopathic fever is peculiar to the human kind.' It may be added, that processes of an inflammatory nature are fitted for repairing parts that have their functions interrupted or their structure injured; but the effects of typhous fever have no such power."

Thus far the evidence which I have adduced and referred to in the foregoing extracts, has chiefly regarded negative proof that signs of inflammation are not uniformly or generally found on minute dissection of those who die of typhous fever, as asserted by those who attribute it to inflammation;—extracts which I would willingly extend, in order to describe more fully the process by which I have endeavoured to come to a right decision on a question, which I believe involves the health and lives of mankind more than any other science; and by stating, as far as my prescribed limits admit, all the circumstances of the case, I wish to enable others to exercise their own judgment, when called on to return a verdict so fearfully important to their fellows.

In tracing the further steps of my inquiries, I mean to illustrate my opinions by extracts from my previous publications, tending to show not only that fever is not essentially derived from disorganisation of the solids, but that its cause, as well as the alterations of structure, may be often traced to originate in the blood, or in the fluids from which it is derived.

With such views, I stated, in my Report for 1820 and 1821, the remarkable coincidence found between the symptoms and appearances after death of the fatal cases which occurred in 1818, when famine excited an extraordinary degree of fever in this country, and those poisonous effects related by Dr. Kerner, to have followed the use of certain unwholesome animal substances at Wirtemburgh;* and I shall repeat the extract I then gave from his work, premising that he also found the whole system of the ganglionic and cerebral nerves generally affected; a circumstance so frequently observed by Dr. Robert Reid, in the dissections of those bodies he examined after death from the fever of 1818, that he supposed that system to be the chief seat of the disease. The similitude between the symptoms to those fatal cases will, I think, be readily recognised by the experienced; and I am, therefore, induced to give the following extract from Dr. Kerner's publication.

"The symptoms of poisoning commonly commence twenty-four hours after the ingestion of the aliment, rarely sooner, and sometimes later: a severe burning pain is then felt in the epigastrium, and vomiting of the sanguineous matter occurs from time to time; the eyes then become fixed, the lids immovable; the pupils are dilated, and remain insensible to the action of light; the patient sees double; the voice is affected, and there is often aphonia, more or less complete; respiration is

---

* *Nouvelles Observations sur les Empoisennements Mortels, qui arrivent si souvent dans le Wirtembourg, par l'usage des Boudins fumes.* Par le Dr. Kener.—Tubingen, 1820. 12 Morn.

impeded; the beating of the heart cannot be felt; there is frequent syncope; the pulse is weaker than natural; the veins of the neck are dilated and prominent; deglutition is extremely difficult, fluids fall into the stomach as into an empty vessel; solid food remains in the œsophagus; all secretion seems suspended. There is an obstinate constipation, or the excreted matters are hard and dry, earthy, and not tinged with bile. The intellectual faculties remain perfect. In some instances the patient becomes irascible; rarely is there insomnia; the appetite often remains; the thirst is great; the integuments insensible; the patient scarcely perceives the impressions of heat and cold; the palm of the hand is hard and coriaceous; the skin, in general, is cold and dry; it is impossible to restore transpiration. Urine is copious; its excretion difficult. The motions are slow from the syncope, which threatens the patient on the slightest effort; but there is no fatigue in the muscles of the back or loins. When death follows, it is at the end of from three to eight days: respiration becomes impeded; the voice is lost; the pulse sinks; and life ceases sometimes after slight convulsive motions, the patient having retained his faculties to the last. In case of recovery, convalescence is very long; a sort of exfoliation often occurs from the mucous membranes. The symptoms vary in different cases, and some are occasionally observed, of which we have not spoken,—as diarrhœa, hydrophobia, delirium, vertigo, atrophy of the testes, &c."

Within the last six, but still more remarkably within the last three years, the many cases of the most exquisite forms of typhous fever, that have succeeded punctures received in dissection, at the various schools of anatomy in London, Dublin, and Edinburgh, have clearly depended on a specific virus introduced through the medium of the animal fluids. I accordingly adverted (in a former Number of this Journal) to facts affording conclusive evidence in favour of the pathology of fever I have adopted. On that occasion, I also attempted to show the analogy between these unfortunate cases, both as to their symptoms and successful treatment, and those of typhus gravior, which have been of late received into the Fever Hospital in Cork-street.

I must not delay longer to enter on the description of the epidemic that committed such havoc on all ranks of our population in the last three years, and by which I proposed to illustrate my pathology of fever, by showing the remarkable coincidence which occurred, during that period, between the intensity of the symptoms of disease and that of the altered condition of the animal fluids. I may be allowed to commence the description with the following extracts from my Report of 1823, which also respect the objects aimed at in those already taken from my Reports of preceding years.

"It may be said, however, that, although dissections may have thus demonstrated that altered structure of parts, or traces of previous inflammation, were not always to be found on examination of the bodies of those who had died of typhous fever, and therefore were not to be deemed its essential characteristics, yet that neither have they established that the fluids are the seat of that disease, or of its being the consequence of changes which primarily took place in them, as implied in the general division of the subject that I have adopted.

**In vitium ducit culpæ fuga, si caret arte.**

"The statements of modern pathologists having been the only plea for setting aside the opinions held by the highest medical authorities from the earliest ages, and down to the very last, with respect to the seat and origin of typhoid pestilential fevers, I deemed it necessary to notice the results of those anatomical researches to which such statements referred, previously to entering on a brief consideration of the nature and seat of the contagious fever of this country, especially under these pestilential characteristics which have designated them within the last year, with a view to inquire how far the principles inculcated by Hippocrates, Sydenham, Meade, and Boerhaave, when treating of pestilential fever at successive periods, are applicable to that which is the immediate object of inquiry.

"The opinion, however, that the animal fluids are primarily and chiefly affected in typhoid fevers by the generation or introduction of some poison, has not been adopted by me solely on authority, or from the analogies just alluded to; but has been the result of the extensive observations on fever which I have had an opportunity of making, as well in this hospital as in private practice, and from having, in different seasons, when pestilential fever prevailed, witnessed those cutaneous eruptions, internal and external hemorrhoidal gangrenes, and even those glandular swellings, by which such fevers, in their most exquisite forms, are characterised. In mixed cases, too, in which the paramount urgency of the inflammatory affection demanded bleeding, as sometimes happens, I have frequently noticed morbid alterations in the appearance of the blood drawn, totally different from that which is supposed to be the effect of inflammation. On the contrary, the crassamentum was imperfectly and loosely coagulated or broken down, the livid fragments mingling in colour as well as in consistence with the opaque, sometimes green or yellow, serum or sanies in which they floated."

"During the rise and progress of the constitutio epidemica that has prevailed in this country now nearly fifteen months, giving to almost all febrile diseases an unusually pestilential character, I witnessed, while examining the blood drawn in different diseases with a view to a particular object, that remarkable changes took place, as the simple fever which accompanied the influenza assumed either a remittent or typhoid character; or, I would prefer saying, as the blood was affected either by successive derangements of its morbid condition excited in the mephitic effects of contagious miasmata: and as such facts appear to me highly corroborative of the opinion now under consideration, I may be excused for treating them somewhat further in detail.

"In the first or catarrhal stage of this epidemic, the blood drawn was generally found of a lighter colour and less consistence than in health, the crassamentum being imperfectly separated. At later stages, the viscera, particularly the lungs and liver, were more frequently engaged. In such cases, the blood drawn was generally buffed; the tunic covering that taken in pulmonary complaints being superficial, cupped, and white; but the buffy coat on blood taken in hepatic complaints was much thicker, darker coloured, and of an evener surface.*

"As the epidemic advanced, the cases of fever more generally wore the characteristics of pestilence: such were the worst forms of cutaneous eruptions, purpura (dark and extensive), external and internal hemorrhages, fetid exhalations, and offensive stench from the alvine discharges. The blood, whether accidentally effused or drawn for the relief of some local congestion, was always found morbidly changed, and, in proportion to the urgency of the foregoing symptoms, dissolved and discoloured, as already mentioned. These observations will be further illustrated by the cases noted in the different stages of the epidemic.

"Being fully persuaded, from such observations, that the nature of that epidemic constitution by which diseases at certain seasons are rendered more rife, and some of them more pestilential, are chiefly to be explained by the pernicious influence of certain conditions of the atmosphere on the animal fluids, particularly on the blood itself, I am the more desirous to record them for the consideration of others.†"

Before I advance further into the description of the epidemic of the last three years, I shall here introduce a Table, which

---

* See Pathological Observations on Dropsy, Purpura, &c.—Dublin, 1823. Page 40—42.

† See my Report of the Fever Hospital and House of Recovery, Cork-street, for 1823; and Tables of the Weather and Admissions, between page 16 and 17 of that Report.

will contrast its extraordinary urgency during this period with its progressive increase during the twenty years preceding, and exhibit the proportion of that increase. This table is similar to those prefixed to my Reports of the Fever Hospital and House of Recovery in Cork-street: as on its columns are inscribed the number of patients admitted, the number of deaths, the average mortality at that institution since it was founded; and, for reasons stated on other occasions, I believe it affords as accurate a scale of the increase of fever, and its severity, in this metropolis, as is possible, without taking into account the vast number of those who would not apply to charitable institutions for relief from this calamity, and of those who were received into the other extensive Fever Hospitals in Dublin. It appears, by this table, that 10,764 patients were admitted in the three years in question, of whom 1,001 died,—a mortality of one in $10\frac{754}{1001}$; and that, in nineteen preceding years, the whole number admitted was 46,561, of whom 2,869 died, giving a mortality of one in $16\frac{607}{2869}$: a fact which cannot fail to excite deep interest respecting the details of the course of the epidemic since the commencement of 1823, and justify my attempt to lay them before the public. These details, however, I find must be again de-

| Years. | Admitted. | Died. | Average. |
|---|---|---|---|
| 1804 | 415 | 29 | 1 in $14\frac{9}{29}$ |
| 1805 | 1024 | 67 | 1 in $15\frac{19}{67}$ |
| 1806 | 1264 | 103 | 1 in $12\frac{28}{103}$ |
| 1807 | 1100 | 92 | 1 in $11\frac{88}{92}$ |
| 1808 | 1071 | 94 | 1 in $11\frac{37}{94}$ |
| 1809 | 1051 | 83 | 1 in $12\frac{65}{83}$ |
| 1810 | 1774 | 154 | 1 in $11\frac{40}{77}$ |
| 1811 | 1472 | 115 | 1 in $12\frac{91}{115}$ |
| 1812 | 2265 | 166 | 1 in $13\frac{107}{160}$ |
| 1813 | 2627 | 164 | 1 in $16\frac{3}{164}$ |
| 1814 | 2329 | 143 | 1 in $16\frac{104}{143}$ |
| 1815 | 3789 | 187 | 1 in $20\frac{40}{178}$ |
| 1816 | 2763 | 173 | 1 in $15\frac{168}{173}$ |
| 1817 | 3682 | 231 | 1 in $15\frac{217}{231}$ |
| 1818 | 7608 | 258 | 1 in $30\frac{126}{258}$ |
| 1819 | 3873 | 224 | 1 in $17\frac{134}{224}$ |
| 1820 | 2974 | 203 | 1 in $14\frac{132}{203}$ |
| 1821 | 2973 | 246 | 1 in $12\frac{76}{246}$ |
| 1822 | 2307 | 137 | 1 in $16\frac{124}{137}$ |
| Total............. | 46,361 | 2869 | 1 in $16\frac{657}{2869}$ |
| 1823 | 2668 | 241 | 1 in $11\frac{17}{251}$ |
| 1824 | 4599 | 327 | 1 in $12\frac{44}{3791}$ |
| 1825 | 3878 | 381 | 1 in $10\frac{68}{381}$ |
| Total for these 3 years | 10,764 | 1001 | 1 in $10\frac{754}{1001}$ |

ferred to another letter, as the general observations which I feel called on to make respecting the whole period embraced by this table will extend as far as the limits usually allowed to articles for the Journal.

Instructive as the facts which this table presents must be to physicians, they should be no less interesting to the political economist; for he may observe, as my occupations have obliged me to do, that this monstrous growth of disease, and also the fearful increase of its severity, have been advancing in measured step with poverty and demoralisation; and that these are excessive only there where the people have nothing to do, or where they want gainful employment. With great justice have writers on the epidemics of this country adverted to many other concurring causes; but, surely, from the uniformity of the phenomena, indicating a corresponding uniformity in the cause, we should chiefly look to that which has coexisted with both. Before now I have had occasion to observe with what direful rapidity disease has advanced, when attended by that wretchedness of both mind and body which want of employment must necessarily beget; and often has it been a subject of amazement to me, that inventions which curtail manual labour, and thus must have deplorably increased that want of employment, should not have been esteemed a chief source of misery in a country whose poverty is so great that she cannot with advantage export, and whose population is so dense that the cultivation of all her natural resources would be necessary to give sufficient occupation to the inhabitants.

I anxiously wish that public attention would turn to the growth of those national evils which have been so direfully remarkable of late years, and more especially as the consistency of my humble evidence might be questioned, because Parliament have been pleased to bestow so little notice upon these evils, so remarkably urgent at the time of the late inquiries into the state of Ireland. It is not for me to conjecture why such subjects were not embraced in these inquiries; but I would be glad that politicians would, in this instance at least, stoop to a suggestion which the practice of medicine affords; as when the physician, finding his treatment unsuccessful, because ill-conceived, retraces his former steps, that he may find the point at which he erred, and thus brought back to the consideration of the true origin and nature of diseases, is taught, by his former mistakes, more effectually to prescribe for them.

For the removal or alleviation of febrile diseases, there has been erected the Fever Hospital and House of Recovery in Cork-street, which has internal and external accommodation of unprecedented extent for the cure of the sick, separate from their supposed infected dwellings, and for the destruction of contagious fomites. Besides this, there are other extensive hospitals through the city for like purposes; whilst, by widening the streets, ventilating, cleansing, and whitewashing the apartments of the poor, and by giving them plentiful supplies of water by fountains erected at their very doors, it was expected that the other causes, which were supposed to concur in producing the calamities in question, might be modified, if not removed; and yet it is too apparent that the source has not been stopped, but that, on the contrary, its produce has greatly exceeded all former bounds, and its effects been even more pernicious than before.

*York-street; April* 21, 1826.

